# Locomotor and discriminative stimulus effects of N-cyclohexyl butylone and N-cyclohexyl methylone

**DOI:** 10.3389/fphar.2026.1823177

**Published:** 2026-05-18

**Authors:** Michael B. Gatch, Shuping Jia, Ritu A. Shetty, Rebecca D. Hill, Nathalie Sumien, Michael J. Forster

**Affiliations:** Department of Pharmacology and Neuroscience, University of North Texas Health Science Center, Fort Worth, TX, United States

**Keywords:** cathinones, cocaine, drug discrimination, MDMA, methamphetamine, psychostimulants, rodents

## Abstract

**Background:**

N-Cyclohexyl butylone and N-cyclohexyl methylone are cathinones found on the street that produce morbidity and mortality, but are not yet legally controlled, and there has been little or no pharmacological testing of these compounds. Both compounds have a large moiety on the N-position that may alter their pharmacological effects.

**Methods:**

Locomotor stimulant effects of N-cyclohexyl butylone and N-cyclohexyl methylone were tested in male Swiss-Webster mice. N-Cyclohexyl butylone and N-cyclohexyl methylone were tested in male Sprague-Dawley rats trained to discriminate methamphetamine (1 mg/kg), cocaine (10 mg/kg) or 3,4-methylenedioxymethamphetamine (MDMA, 1.5 mg/kg) from saline.

**Results:**

Both N-cyclohexyl butylone (ED_50_ = 2.80 mg/kg) and N-cyclohexyl methylone (ED_50_ = 33.46 mg/kg) produced dose- and time-dependent stimulation of locomotor activity that was both less potent and less efficacious than that of methamphetamine (ED_50_ = 0.35 mg/kg). N-Cyclohexyl butylone fully substituted for the discriminative stimulus effects of methamphetamine (ED_50_ = 9.85 mg/kg) and cocaine (ED_50_ = 14.54 mg/kg). N-Cyclohexyl methylone failed to fully substitute for cocaine or methamphetamine up to doses that produced convulsions. Both compounds failed to substitute for MDMA up to doses that suppressed responding.

**Conclusion:**

N-cyclohexyl butylone may produce psychostimulant-like effects adequate to motivate illicit use, whereas the weak psychostimulant-like effects of N-cyclohexyl methylone may suggest a reduced liability for illicit use, but the compound may be more dangerous due to its convulsant effects. The N-cyclohexyl moiety may have contributed to the reduced efficacy of N-cyclohexyl methylone, while the longer alpha-side chain of N-cyclohexyl butylone may have countered the effect of the large N-position moiety.

## 1 Introduction

N-cyclohexyl methylone (cyputylone) and N-cyclohexyl butylone are novel psychoactive substances (NPS) that have been flagged by the US Drug Enforcement Administration (DEA) as potential health hazards in the United States. The Center for Forensic Science Research and Education (CFSRE), which generates quarterly reports on the occurrences of NPS *via* their NPS Discovery program, has issued reports for both N-cyclohexyl methylone and N-cyclohexyl butylone (CFSRE, 2022a; 2022b). Increasing use of N-cyclohexyl methylone since 2021 has been reported (Seibert et al., 2025), and a toxicology assay has been developed (Fogarty et al., 2025) to improve detection. Currently, there are no data on their pharmacological effects.

**FIGURE 1:**
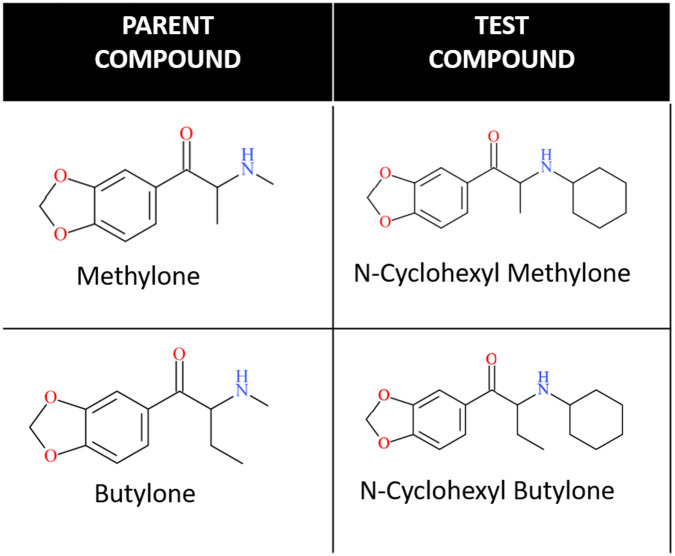
Chemical Structures of test compounds. The illustrations show the chemical structures of the two test compounds along with their respective parent compounds.

N-cyclohexyl methylone and N-cyclohexyl butylone are structurally related to methylone and butylone, which are cathinones with a 3,4-methylenedioxy substitution (Figure 1). The difference between methylone and butylone is that methylone has one carbon at the alpha position, whereas butylone has a two-carbon chain on the alpha position. N-cyclohexyl methylone and N-cyclohexyl butylone differ from the parent compounds by a N-cyclohexane ring substitution.

The parent compounds, methylone and butylone, were two of the earliest synthetic cathinones to be widely misused (Prosser and Nelson, 2012; Simmons et al., 2018). Both produce cocaine-, methamphetamine- and 3,4-methylenedioxymethamphetamine (MDMA)-like discriminative stimulus effects in rats (Gatch et al., 2013; Dolan et al., 2018) and are self-administered (Arde and Taffe, 2017; Dolan et al., 2018; Schindler et al., 2016). Both are active at dopamine transporters (DAT), norepinephrine transporters (NET) and serotonin transporters (SERT), with more SERT activity than some cathinones (Eshleman et al., 2013; López-Arnau et al., 2012), which allows them to have MDMA-like discriminative stimulus effects as well as psychostimulant-like effects. These findings indicate that methylone and butylone have substantial abuse liability and the potential to be used in place of psychostimulants like cocaine, methamphetamine or MDMA.

Other modifications to the methylone and butylone parent molecules have been made, for example, dimethylone and dibutylone. Dimethylone and dibutylone have two N-carbons instead of just one like methylone and butylone. Increases in the size of N-terminal substitutions seem to increase potency/efficacy (Duart-Castells et al., 2021; Nadal-Gratacós et al., 2021) as does increasing the length of the alpha-side chain (e.g., Kolanos et al., 2015; Nadal-Gratacós et al., 2023). This hypothesis was confirmed in the case of dimethylone and dibutyline by findings that dibutylone fully substituted for the discriminative stimulus effects of cocaine, methamphetamine and MDMA, whereas dimethylone (with a shorter-alpha side chain) only partially substituted for cocaine and methamphetamine but fully substituted for MDMA (Gatch et al., 2019). Dibutylone is self-administered (Lai et al., 2022). No self-administration studies with dimethylone have currently been published.

However, given that N-cyclohexyl methylone and N-cyclohexyl butylone have a much larger moiety attached to the N-carbon position than dimethylone and dibutylone, a further decline in psychostimulant efficacy may be observed. An earlier study did report that adding a piperidine (5 carbons) at the N position resulted in a marked decrease in potency and efficacy at producing locomotor stimulant effects (Duart-Castells et al., 2021), suggesting that making the N-position additions too bulky limits receptor interactions. Behavioral testing of N-cyclohexyl methylone and N-cyclohexyl butylone was conducted to determine whether they have psychostimulant or entactogenic effects which may encourage illicit use. Locomotor activity was tested to confirm psychostimulant effects and to determine effective dose ranges and time course. Subjective effects were modeled by testing for discriminative stimulus effects in methamphetamine-, cocaine- and MDMA-trained rats.

## 2 Materials and methods

### 2.1 Subjects

Male Swiss-Webster mice (n = 144) and Sprague-Dawley rats (n = 44) were purchased from Envigo (Indianapolis, IN) at approximately 2 months of age. All animals were allowed to acclimatize in the vivarium for about 2 weeks prior to behavioral testing. Mice were group housed (n = 4/cage) and allowed free access to food. Rats were housed individually, and weight clamped at 320-350 g by limiting their access to food. All rats received a total of approximately 15 g of food per day including the food pellets they received during operant sessions. Animals were maintained on a 12/12 light/dark cycle (lights on at 7:00 a.m.) and had free access to water. All housing and procedures were approved by the University of North Texas Health Science Center Institutional Animal Care and Use Committee.

### 2.2 Locomotor activity

The study was conducted using Digiscan (Omnitech Electronics, Columbus, OH) locomotor activity testing chambers as previously described (Gatch et al., 2025). Separate groups of 8 mice were injected *via* the intraperitoneal route with either vehicle (0.9% saline) or a dose of methamphetamine (0.1, 0.25, 0.5, 1, 2, or 4 mg/kg), N-cyclohexyl butylone (1, 2.5, 5, 10, or 25 mg/kg), N-cyclohexyl methylone (1, 2.5, 5, 10, 25, or 50 mg/kg). Each mouse was then placed in the locomotor activity testing apparatus. Each compound was tested with a separate group of vehicle controls. Horizontal activity (photocell beam interruption) was measured for 6 h and recorded within 10-min periods. Testing of the cathinone compounds started at 1 mg/kg.

### 2.3 Discrimination procedures

A two-lever choice methodology was used to train separate groups of rats for methamphetamine (1 mg/kg) *versus* vehicle, or cocaine (10 mg/kg) *versus* vehicle discrimination using apparatus described previously (Gatch et al., 2025). Adult male Sprague-Dawley rats were trained to discriminate either methamphetamine (1 mg/kg, i.p., 10-min pretreatment), cocaine (10 mg/kg, i.p., 10-min pretreatment), or MDMA (1.5 mg/kg, i.p., 15-min pretreatment) from 0.9% saline using a FR 10 schedule of food reinforcement (45 mg food pellets; Bio-Serve, Frenchtown, NJ) with a two-lever choice procedure. A reinforcer was available for every 10 responses on a designated injection-appropriate lever. The rats received approximately 60 of these 20-min sessions before they were used in tests for substitution of the experimental compounds. Rats were used in testing once they had achieved 9 of 10 sessions at 85% injection-appropriate responding for both the first reinforcer and total session. The training sessions occurred on separate days in a double alternating fashion (drug-drug-saline-saline-drug, *etc.*). After completion of the training phase, substitution tests were introduced into the training schedule such that at least one saline and one drug session occurred between each test (drug-saline-test-saline-drug-test-drug; *etc.*). The substitution tests occurred only if the rats had achieved 85% injection-appropriate responding on the two prior training sessions. Standard behavior-testing chambers (Coulbourn Instruments, Allentown, PA, Model E10-10) connected to IBM-PC compatible computers *via* LVB interfaces (Med Associates, St. Albans, VT) were used for testing. N-cyclohexyl butylone (1, 2.5, 5, 10, 25, or 50 mg/kg) and N-cyclohexyl methylone (1, 2.5, 5, or 10 mg/kg) were tested for substitution in methamphetamine-trained rats (n = 9), cocaine-trained rats (n = 6), and MDMA-trained rats (n = 6). Test compounds were administered 15 min prior to testing.

### 2.4 Drugs

(+)-Methamphetamine hydrochloride (HCl), (-)-cocaine HCl and (±)-methylenedioxy-methamphetamine HCl were supplied by the National Institute on Drug Abuse Drug Supply Program. N-cyclohexyl butylone HCl, and N-cyclohexyl methylone HCl were supplied by the Drug Enforcement Administration Special Testing Laboratory. Optically active cathinones were provided as racemates. The test compounds were dissolved in saline. N-cyclohexyl methylone solutions were acidic (pH approximately 5.5) and were buffered (using 0.1 M NaOH) prior to injection.

### 2.5 Data analysis

Dose-response analysis was performed on averaged activity data from the 30-min period when maximal stimulation of locomotor activity first appeared at the lowest effective dose, and those data were considered in a one-way analysis of variance (ANOVA) followed by planned comparisons against the vehicle control group. The maximal effect was defined as the ambulation counts at the dose yielding the largest stimulant effect on the dose-response curve, and the cathinones were compared for efficacy using a one-way ANOVA. Statistical significance was set at p < 0.05.

For the drug discrimination data, the mean percentage of drug-lever responses and response rate were calculated, and these measures were plotted against dose using a log scale. The percentage of drug-lever responding data were not considered if less than 3 rats completed the first fixed ratio. Full substitution was defined as mean percent drug-lever responding ≥ 80%. Rates of responding were expressed as the number of responses divided by the total session time (in seconds). Response rate data were analyzed by one-way repeated measures ANOVA. The mean response rate after each dose was compared to that of the vehicle control value using planned comparisons.

Linear regressions of the linear portion of the dose-response data were performed on the pooled data for each compound, and potencies were calculated (ED50 ± standard error of the mean (SEM)) using OriginGraph (OriginLab Corporation, Northampton, MA). Locomotor activity potencies were based on 50% of the maximum effect of the given drug. Drug discrimination potencies were based on 50% drug-appropriate responding. Locomotor stimulant maximal effects were compared using one-way ANOVAs. Individual differences were compared using the Tukey test.

## 3 Results

### 3.1 Locomotor activity

**FIGURE 2:**
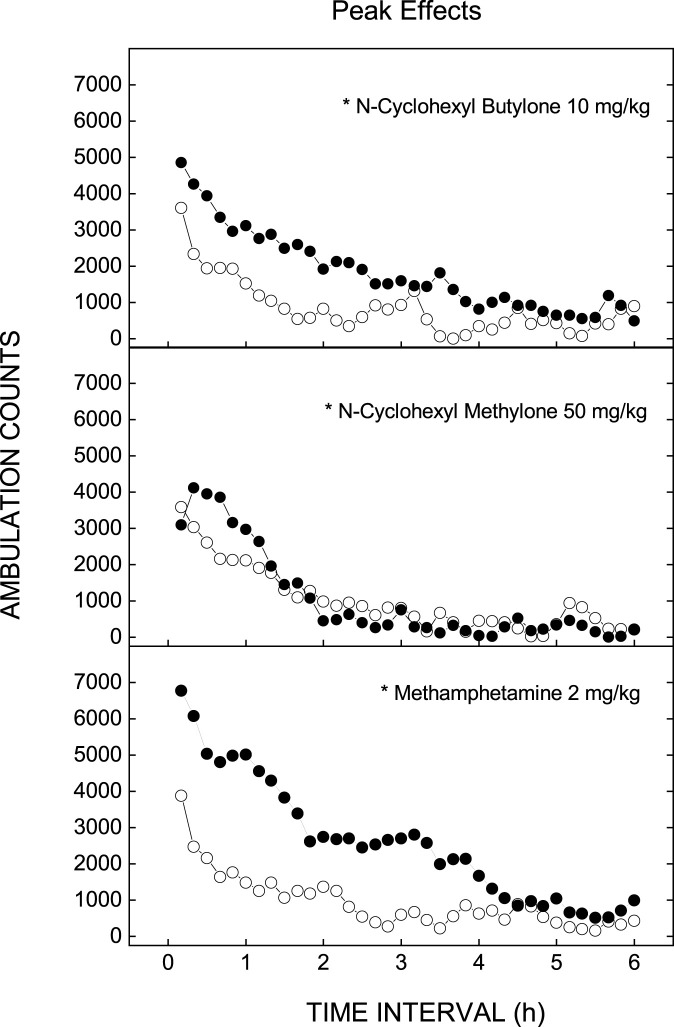
Locomotor activity time courses of doses with maximal effects.

**FIGURE 3:**
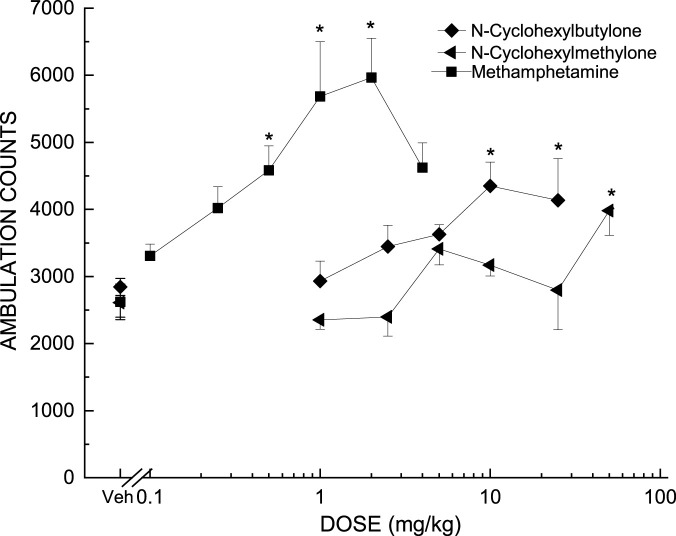
Locomotor Activity Dose Effect. Each data point represents the mean ambulation counts/10min ± SEM, measured during the earliest 30-min period of maximal effect, plotted as a function of dose. Each dose was tested in independent groups of 8 mice per dose (unless otherwise specified). Dose groups that differed significantly from vehicle treated controls during the defined effect period are indicated by asterisks (*p* < 0.05).

Time courses of the doses of each test compound that produced maximal effect are shown in Figure 2. Dose effects of the locomotor stimulant effects of the test compounds are shown in Figure 3. (+)-Methamphetamine produced time- and dose-dependent increases in locomotor activity to a peak effect of 5,967 ± 585 counts following 2 mg/kg Dose *F* (6,49) = 12.553, *p<*0.001; Time *F* (35,1715) = 70.0, *p<*0.001; Time X Dose Interaction *F* (210,1715) = 3.796, *p<*0.001. Locomotor stimulant effects occurred within 10 min and lasted 4 h. A higher dose (4 mg/kg) decreased locomotor activity. An ED_50_ of 0.35 mg/kg was determined based upon a linear regression against log10 doses from 0.1 to 2 mg/kg (+)-methamphetamine (Table 1). A one-way analysis of variance indicated a significant effect of Treatment *F*(6,49) = 6.512, *p <* 0.001, and planned comparisons (*a priori* contrast) against the vehicle group showed a significant effect for the 0.5, 1, and 2 mg/kg doses (*p*s < 0.05 denoted on Figures 2, 3 with an asterisk).

**TABLE 1:** Potencies (ED_50_ in mg/kg ± 95% confidence interval) of the test compounds in drug discrimination and locomotor activity testing.

Test compound	Methamphetamine drug discrimination	Cocaine drug discrimination	MDMA drug discrimination	Locomotor activity
N-Cyclohexyl methylone	NA	NA	NA	33.46 (5.49–203.8)
N-Cyclohexyl butylone	9.85 (1.33–72.9)	14.54 (2.70–76.75	NA	2.80 (0.16–49.3)
Methamphetamine	0.20 (0.02–1.99)	--	--	0.35 (0.017–6.93)
Cocaine	--	2.11 (0.07–63.79)	--	--
MDMA	--	--	0.83 (0.11 – 5.95)	--

N-cyclohexyl butylone produced time- and dose-dependent increases in locomotor activity following 10 and 25 mg/kg Dose *F* (5,42) = 2.935, *p = 0*.023; Time *F* (35,1470) = 57.913, *p<*0.001; Time X Dose Interaction *F* (175,1470) = 1.498, *p<*0.001. Locomotor stimulant effects occurred within 10 min and lasted 3 h. The peak effect of 4,350 ± 356 counts occurred following 10 mg/kg [*F* (5,42) = 3.364, *p* = 0.012]. An ED_50_ of 2.80 mg/kg was determined based upon a linear regression against log10 doses from 1 to 10 mg/kg N-cyclohexyl butylone.

N-cyclohexyl methylone produced time- and dose-dependent increases in locomotor activity following 5 mg/kg Dose *F* (6,49) = 4.822, *p =* 0.001; Time *F* (35,11,715) = 93.88, *p <* 0.001; Time X Dose Interaction *F* (210,1715) = 2.043, *p <* 0.001. The peak effect of 3,981 ± 369 counts occurred following 50 mg/kg [*F* (6,49) = 3.463, *p* = 0.006]. An ED_50_ of 33.46 mg/kg was determined based upon a linear regression against log10 doses from 25 to 50 mg/kg N-cyclohexyl methylone. The peak effects of N-cyclohexyl butylone and N-cyclohexyl methylone were less than that of (+)-methamphetamine [*F* (2,21) = 5.531, *p* = 0.012] as shown in Table 2.

**TABLE 2:** Peak effects^a^ of the test compounds in drug discrimination (percent drug-appropriate responding ± standard error) and locomotor activity testing (ambulation counts ± standard error).

Test compound	Methamphetamine drug discrimination	Cocaine drug discrimination	MDMA drug discrimination	Locomotor activity
N-Cyclohexyl methylone	55 ± 17	13 ± 12	33 ± 21	3,981 ± 369
N-Cyclohexyl butylone	89 ± 10	83 ± 17	1 ± 1	4,350 ± 356
Methamphetamine	99 ± 0.2	--	--	5,967 ± 585
Cocaine	--	88 ± 8	--	--
MDMA	--	--	85 ± 10	--

^a^ Full substitution was defined as mean percent drug-lever responding ≥ 80%.

The relative efficacy of N-cyclohexyl butylone to (+)-methamphetamine was 73% ((Emax test compound/Emax reference) compound*100) and the relative efficacy of N-cyclohexyl methylone to (+)-methamphetamine was 67%. N-cyclohexyl butylone was 8-fold less potent than methamphetamine and N-cyclohexyl methylone was 96-fold less potent than methamphetamine.

### 3.2 Drug discrimination

**FIGURE 4:**
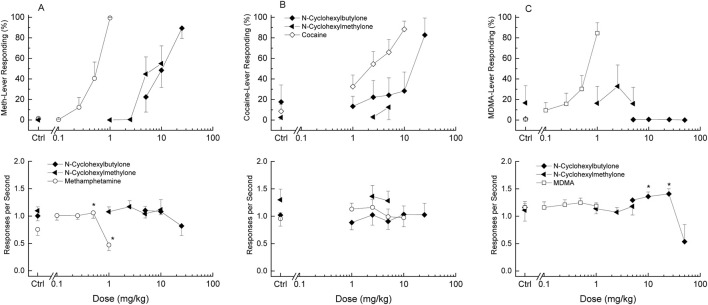
Drug Discrimination Dose Effect. The substitution effects of each test compound in methamphetamine- (Meth, **(A)**), cocaine- **(B)**, and MDMA-trained rats **(C)**. The upper panels depict the percentage of total responses on the drug-appropriate lever. The bottom panels show response rates expressed as responses per second. N = 6 unless otherwise specified. “Ctrl” denotes the vehicle control values. In the response rate panels, doses that differed significantly from vehicle are marked with asterisk (*p* < 0.05).

Methamphetamine (ED_50_ = 0.20 mg/kg) substituted fully for the training dose of 1 mg/kg methamphetamine (Figure 4, Panel A). Response rate was decreased to 62% of vehicle control following 1 mg/kg F (5,45) = 7.767, p < 0.001. Cocaine (ED_50_ = 2.11 mg/kg) substituted fully for the training dose of 10 mg/kg cocaine (Figure 4, Panel B). Response rate failed to show significant change from vehicle control following 1 to 10 mg/kg cocaine F (4,56) = 0.86, p = 0.491. MDMA (ED_50_ = 0.83 mg/kg) substituted fully for the training dose of 1.5 mg/kg MDMA (Figure 4, Panel C). Response rate was not changed at the doses tested F (4,48) = 0.51, p = 0.728. Potencies are shown in Table 1 and peak effects are shown in Table 2.

N-Cyclohexyl butylone (ED_50_ = 9.85 mg/kg) substituted fully for the training dose of 1 mg/kg methamphetamine in a group of nine rats (Figure 4). Response rate was not changed at the doses tested F (3,24) = 2.234, p = 0.11. N-cyclohexyl butylone (ED_50_ = 14.54 mg/kg) fully substituted for the discriminative stimulus effects of 10 mg/kg cocaine in a group of 6 rats. Response rate was not changed at the doses tested *F* (5,25) = 0.55, *p* = 0.735. Within the dose range of 5 to 50 mg/kg, N-cyclohexyl butylone failed to substitute for the discriminative stimulus effects produced by 1.5 mg/kg of MDMA in a group of 6 rats. Peak MDMA-appropriate responding was 1% ± 1%. Response rate was increased following 10 and 25 mg/kg N-cyclohexyl butylone *F* (4,12) = 4.45, *p* = 0.020. Due to the limited supply of N-cyclohexyl butylone, fewer than six rats were tested following the 10, 25 and 50 mg/kg doses. Methamphetamine was 50-fold more potent than N-cyclohexyl butylone and cocaine was 7-fold more potent.

N-Cyclohexyl methylone, tested in doses from 1 to 10 mg/kg, produced peak methamphetamine-appropriate responding of 55% ± 17% in a group of 9 rats. Response rate was not changed following 1 to 10 mg/kg N-cyclohexyl methylone F (4,28) = 0.714, p = 0.589. Clonic convulsions were observed in one of eight rats following 10 mg/kg N-cyclohexyl methylone. Only eight rats were tested following 10 mg/kg due to the adverse effects. Within the dose range of 2.5 to 5 mg/kg, N-cyclohexyl methylone failed to substitute for the discriminative stimulus effects produced by 10 mg/kg of cocaine in a group of 6 rats. Higher doses were not tested due to the convulsions observed at 10 mg/kg in the methamphetamine study. Peak cocaine-appropriate responding was 13% ± 12%. Response rate was not changed *F* (2,10) = 0.28, *p* = 0.764. When tested from 1 to 5 mg/kg, N-cyclohexyl methylone failed to substitute for MDMA in a group of 6 rats. Peak MDMA-appropriate responding was 33% ± 21%. Higher doses were not tested due to the convulsions seen at 10 mg/kg in the methamphetamine study. A one-way, repeated measures analysis of variance conducted on response rate for the total session failed to indicate a significant overall effect *F* (3,15) = 0.21, *p* = 0.888.

## 4 Discussion

N-cyclohexyl butylone and N-cyclohexyl methylone are cathinones based on butylone and methylone with a 3,4-methylenedioxy substitution on the phenyl ring and a N-cyclohexane ring substitution. The structural difference between the two compounds is that N-cyclohexyl methylone has one carbon attached at the alpha position, whereas N-cyclohexyl butylone has a two carbon chain on the alpha position. In the present study, N-cyclohexyl butylone and N-cyclohexyl methylone were weak psychostimulants, increasing locomotor activity to a lesser extent than methamphetamine. Both compounds were less potent and less efficacious than methamphetamine. N-cyclohexyl butylone was 8-fold less potent and N-cyclohexyl methylone was nearly 100-fold less potent than methamphetamine. N-Cyclohexyl methylone was 12-fold less potent than N-cyclohexyl butylone, but both compounds had similar efficacy in the locomotor activity assay, producing similar maximal ambulation counts. The ED_50_ for N-cyclohexyl methylone was approximated due to the non-linear nature of the data. Motor activity increased at 5 mg/kg, N-cyclohexyl methylone, then decreased to baseline levels at 25 mg/kg, and again increasing to a peak at 50 mg/kg. The reliability of the ED_50_ for N-cyclohexyl methylone was very low due to the lack of linearity, as can been seen by the wide 95% confidence interval in Table 1. Such inconsistent effects are common with weak compounds and it is apparent from Figure 3 that N-cyclohexyl methylone was less potent than N-cyclohexyl butylone and much less potent than methamphetamine.

The discriminative stimulus effects of N-cyclohexyl butylone and N-cyclohexyl methylone were tested in methamphetamine-, cocaine- and MDMA-trained rats. N-Cyclohexyl butylone fully substituted for methamphetamine and cocaine. N-Cyclohexyl methylone produced about half-maximal effects in methamphetamine-trained rats and failed to substitute for cocaine. Neither compound produced MDMA-like discriminative stimulus effects.

N-Cyclohexyl methylone produced lower levels of methamphetamine-, cocaine- and MDMA-appropriate responding than did dimethylone, which produced submaximal cocaine-like responding, and fully substituted for methamphetamine and MDMA in a previous study (Gatch et al., 2019). However, the peak effects of dimethylone were observed at 25 mg/kg, whereas only 5 to 10 mg/kg of N-cyclohexyl methylone was tested in the present study due to the convulsions observed at 10 mg/kg. It is possible that N-cyclohexyl methylone may have produced higher levels of methamphetamine-, cocaine- and MDMA-appropriate responding if higher doses could have been tested. Dibutylone fully substituted for methamphetamine and cocaine at 5 mg/kg, and for MDMA at 50 mg/kg (Gatch et al., 2019), whereas in the present study, N-cyclohexyl butylone was less potent, substituting for methamphetamine and cocaine at 25 mg/kg and failed to substitute for MDMA up to 50 mg/kg, which suppressed respond rate. Higher doses of N-cyclohexyl butylone could not be tested due to the rate suppression, so it was not possible to determine whether it could have substituted for MDMA at higher doses. Even if it had, N-cyclohexyl butylone would have been less potent than dibutylone.

Methylone, the parent compound for N-cyclohexyl methylone, is fundamentally the cathinone form of MDMA, differing from MDMA only in the presence of the β ketone. Not surprisingly, methylone’s *in vitro* pharmacological effects are similar to those of MDMA (Baumann et al., 2012; Poyatos et al., 2023). Looking at structural commonalties from another direction, methylone is a version of methcathinone with a 3,4-methylenedioxy substitution. The 3,4-methylenedioxy substitution makes cathinone compounds more serotonergic whereas increasing the length of alpha-side chain makes cathinone compounds more dopaminergic (e.g., Kolanos et al., 2015; Nadal-Gratacós et al., 2023) and the DAT/SERT ratio predicts reinforcement efficacy (Dolan et al., 2018; Gannon et al., 2018; Negus and Banks, 2017). So again, not surprisingly, methylone is more serotonergic and less dopaminergic than methcathinone (Baumann et al., 2012; Eshleman et al., 2013; Wakeford et al., 2021).

Butylone is the cathinone form of MBDB (N-methyl-1,3-benzodioxolylbutanamine) another entactogen. MBDB’s *in vitro* effects are similar to those of MDMA, producing a strong release of serotonin, less NE release and no dopamine release, as well as inhibition of serotonin and noradrenaline re-uptake (Aerts et al., 2000; Nagai et al., 2007). Similarly, buphedrone, also known as α-methylamino-butyrophenone (MABP), shares the phenylamine structure of butylone, but without the 3,4-methylenedioxy substitution. Buphredrone preferentially inhibited uptake of NE and DA and also released NE (Simmler et al., 2014).

In behavioral testing, methylone and butylone were both cocaine- and methamphetamine-like, producing full substitution (Gatch et al., 2013; Dolan et al., 2018). Both compounds were also MDMA-like, fully substituting for MDMA (Dolan et al., 2018). Methylone fully substituted for cocaine in rhesus monkeys (Smith et al., 2017), and substituted for MDMA in squirrel monkeys but failed to fully substitute for methamphetamine (Wakeford et al., 2021). Interestingly, pentylone has one more alpha carbon on the alpha side chain than butylone (for a total of three), and has no MDMA-like efficacy, being strongly dopaminergic (Dolan et al., 2018). In addition, methylone and butylone were both locomotor activity stimulants in mice, with efficacy not different from methamphetamine (López-Arnau et al., 2012; Marusich et al., 2012), although potency was 4- to 7- fold less than that of methamphetamine (Gatch et al., 2013). Buphedrone produced a conditioned place preference and self-administration, and increased locomotor activity as expected from a NET/DAT uptake inhibitor (Oh et al., 2018).

A possibility is that the large N-cyclohexyl group was responsible for the decreased efficacy in the drug discrimination assays. At first glance, evidence does not appear to support this hypothesis, since earlier work has indicated that increased size of substitutions at the N position increase potency; but there may be a limit to the size that will do so (Duart-Castells et al., 2021; Nadal-Gratacos et al., 2021). Dimethylone and dibutylone have two N-carbons instead of just one like methylone and butylone, but still have much smaller N-position moieties than the N-cyclohexyl compounds. Dibutylone fully substituted for cocaine, methamphetamine and MDMA in rats; whereas dimethylone produced submaximal cocaine-like responding, but fully substituted for methamphetamine and MDMA (Gatch et al., 2019). Both were locomotor activity stimulants, but were less efficacious and 10- to 20-fold less potent than methamphetamine (Gatch et al., 2019; Glatfelter et al., 2021). These results are not surprising since dibutylone was selective for DAT with little or no SERT activity (Eshleman et al., 2018), whereas dimethylone blocked DAT but also weakly blocked SERT currents (Solis, 2017).

Other cathinones with the 3,4-methylenedioxy ring and the N-ethyl substitution, such as ethylone and N-ethylpentylone also produced full substitution for cocaine and methamphetamine (Gatch et al., 2017; Gatch et al., 2019), as did dipentylone, which has two N-carbons like dimethylone and dibutylone (Gatch et al., 2021). The N-pyrrolidine substitution, which is comprised of a ring of four carbons on the nitrogen atom is well-known to increase efficacy at dopamine transporters with concomitant increases in reinforcing effects (Gannon et al., 2018; Nadal-Gratacos et al., 2024). Compounds with both the 3,4-methylenedioxy ring and the N-pyrrolidine substitution, such as 3′,4′-methylenedioxy-α-pyrrolidinobutyrophenone (MD-PBP), 3,4-methylenedioxypyrovalerone (MDPV) and 3,4-methylenedioxy-α-pyrrolidinohexanophenone (3,4-MD-α-PHP), all fully substitute for methamphetamine, although MD-PBP produced only 67% cocaine-appropriate responding (Fantegrossi et al., 2013; Gatch et al., 2013; Gatch et al., 2017; Shetty et al., 2023). However, N-butylhexedrone, which has a four-carbon chain at the N position, failed to substitute for methamphetamine and produced only 67% cocaine-appropriate responding (Gatch et al., 2025), which may indicate that large enough moieties at the N-position may decrease efficacy.

One study investigated N-ethyl substitutions with and without phenyl-ring additions: N-ethyl-pentedrone (NEPD), N-ethyl-pentylone (NEP), and 4-methyl-ethylaminopentedrone (4-MeAP) (Nadal-Gratacos et al., 2021). Increased locomotor stimulant effects were observed compared to the analogs with a N-methyl substitution in that study. Another study systematically increased N-terminal size while maintaining the same alpha-side chain and phenyl-ring structures (Duart-Castells et al., 2021). In that study, adding length to the N-chain or a pyrrolidine ring (4 carbons) increased efficacy and potency of locomotor stimulant effects. Adding a double chain (diethyl) further increased efficacy, but decreased potency. Adding a piperidine ring (5 carbons) decreased both potency and efficacy of the locomotor stimulant effects in that study, similar to the N-cyclohexyl compounds (6 carbons) in the present study.

In summary, neither N-cyclohexyl methylone nor N-cyclohexyl butylone were MDMA-like despite having the 3,4-methylenedioxy moiety. Based on these findings, both compounds should have low activity at SERT. Both compounds were weak locomotor stimulants, so should have some activity at DAT and possible NET. N-cyclohexyl butylone was strongly cocaine- and methamphetamine-like, but N-cyclohexyl methylone was not cocaine-like and only weakly methamphetatmine-like, so it would be expected that N-cyclohexyl butylone would have stronger effects at DAT than N-cyclohexyl methylone. It is possible that the bulky N-cyclohexyl moiety reduces efficacy/potency at DAT, which is partially overcome in N-cyclohexyl butylone by having a longer alpha side chain (e.g., Kolanos et al., 2015; Nadal-Gratacós et al., 2023). Testing other N-cyclohexyl cathinones with longer alpha side chains will be necessary to confirm this hypothesis. In addition, such bulky substitutions may also alter absorption kinetics or even brain penetrance. Pharmacokinetic studies will be necessary to test these hypotheses. Finally, N-cyclohexyl butylone may have a potential for misuse similar to other cathinone compounds such as butylone, but N-cyclohexyl methylone may not be likely to be misused intentionally. Place preference and/or self-administration studies are necessary to confirm the abuse potential of these compounds, although drug discrimination studies are excellent predictors (Horton et al., 2013).

## Data Availability

The raw data supporting the conclusions of this article will be made available by the authors, without undue reservation.
